# Supplementary figures and images for: p300/CBP is an essential driver of pathogenic enhancer activity and gene expression in Ewing sarcoma
Source: EMBO Rep. 2025 Sep 1;26(19):4766–93. doi: 10.1038/s44319-025-00552-z (PMC12508431; doi:10.1038/s44319-025-00552-z)

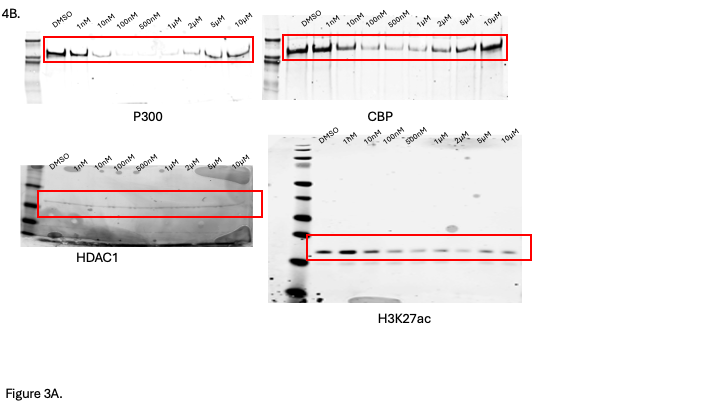

Supplement: Supplementary file 14 — Source data Fig. 3 [file 44319_2025_552_MOESM14_ESM.zip › Figure_3A_Western_blots_1.tiff]

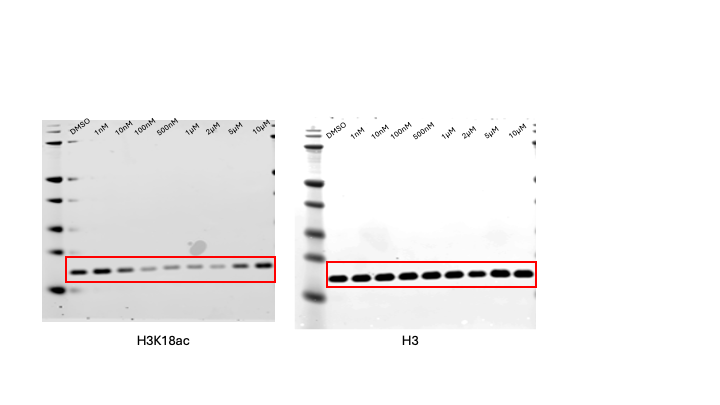

Supplement: Supplementary file 14 — Source data Fig. 3 [file 44319_2025_552_MOESM14_ESM.zip › Figure_3A_Western_blots_2.tiff]

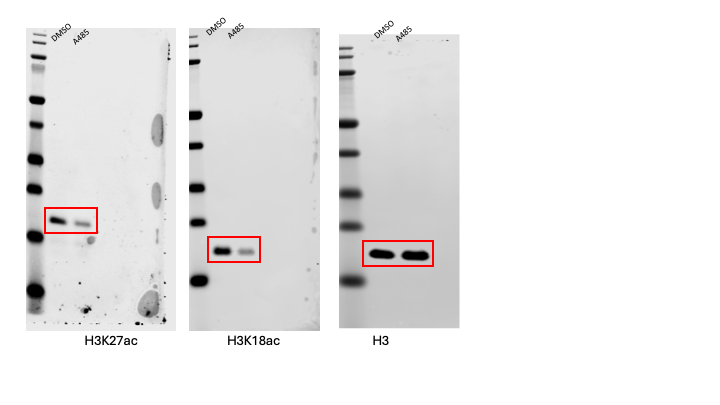

Supplement: Supplementary file 14 — Source data Fig. 3 [file 44319_2025_552_MOESM14_ESM.zip › Figure_3B_Western_blots.tiff]

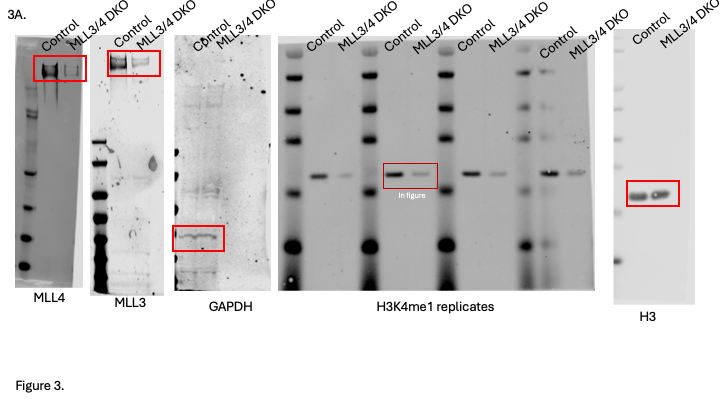

Supplement: Supplementary file 14 — Source data Fig. 3 [file 44319_2025_552_MOESM14_ESM.zip › Figure_3K_Western_blots.tiff]

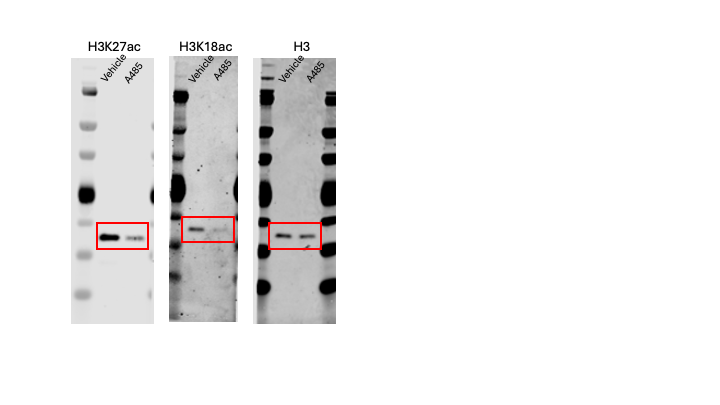

Supplement: Supplementary file 17 — Source data Fig. 6 [file 44319_2025_552_MOESM17_ESM.zip › Figure_6E_-_Western_blot.tiff]
