# Supplementary material for: p300/CBP is an essential driver of pathogenic enhancer activity and gene expression in Ewing sarcoma
Source: EMBO Rep. 2025 Sep 1;26(19):4766–93. doi: 10.1038/s44319-025-00552-z (PMC12508431; doi:10.1038/s44319-025-00552-z)
Supplement: Supplementary file 18 — Expanded View Figures [file 44319_2025_552_MOESM18_ESM.pdf]

Expanded View Figures

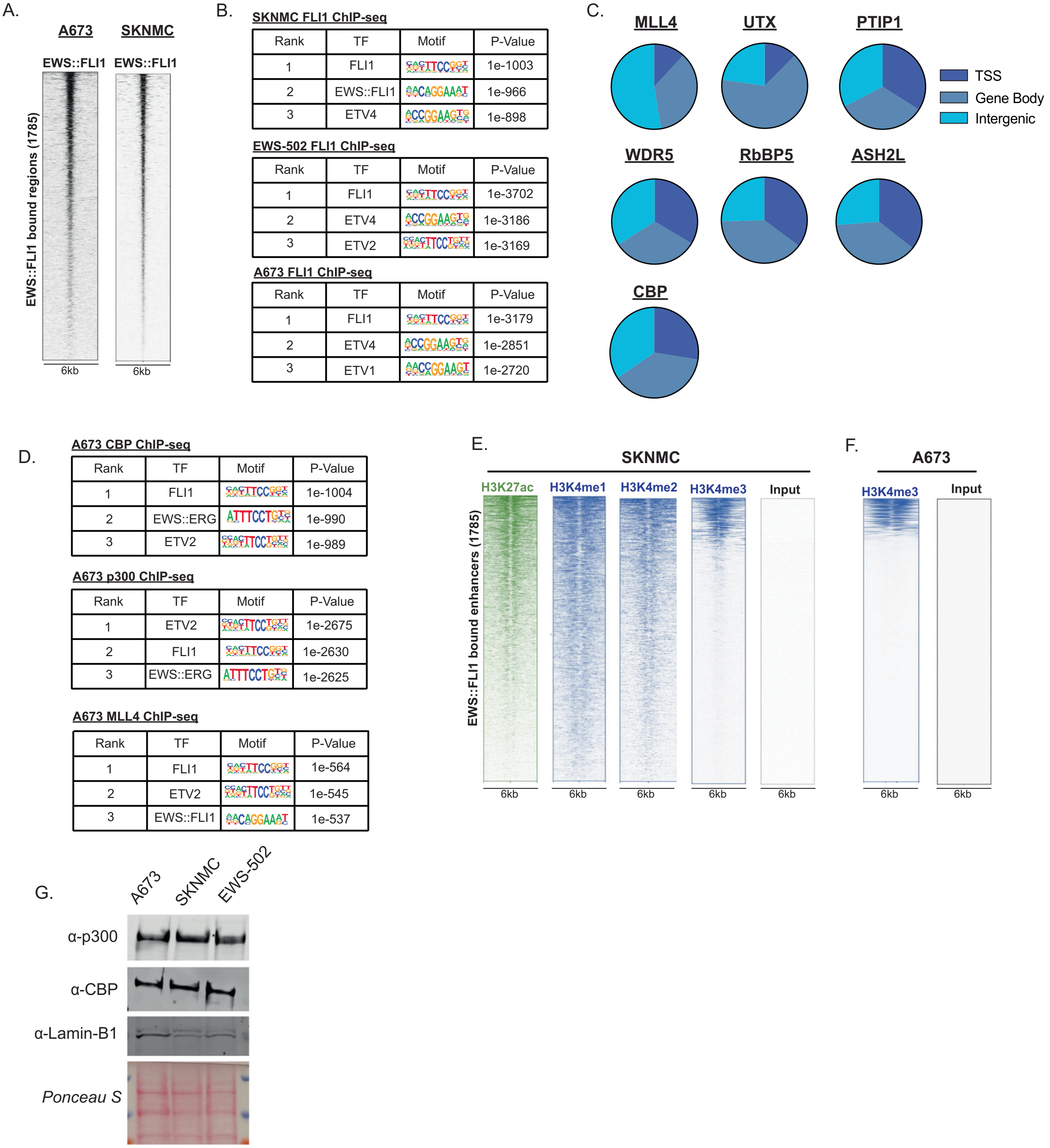

Figure EV1. p300/CBP occupy EWS::FLI1 enhancer regions and mirror EWS::FLI1 binding intensity.

(**A**) Tornado plots of FLI1 ChIP-seq in A673 and SKNMC cell lines at 1785 defined EWS::FLI1 enhancer regions. (**B**) Homer Motif analysis showing top 3 enriched motifs in FLI1 ChIP-seq in SKNMC, EWS-502 and A673 cell lines. (**C**) Genomic location of ChIP-seq peaks called using MACS2 of chromatin factors in A673 cells. (**D**) Homer Motif analysis showing top 3 enriched motifs in CBP, p300 and MLL4 ChIP-seq in A673 cells. (**E**) Tornado plots showing histone modification ChIP-seq signal in SKNMC cells at EWS::FLI1 enhancer regions. Signal compared to input. (**F**) Tornado plots for H3K4me3 ChIP-seq and associated input in A673 cells at EWS::FLI1 enhancer regions. (**G**) Western blot analysis of p300 and CBP in steady-state A673, SKNMC and EWS-502 cells.

 © The Author(s)

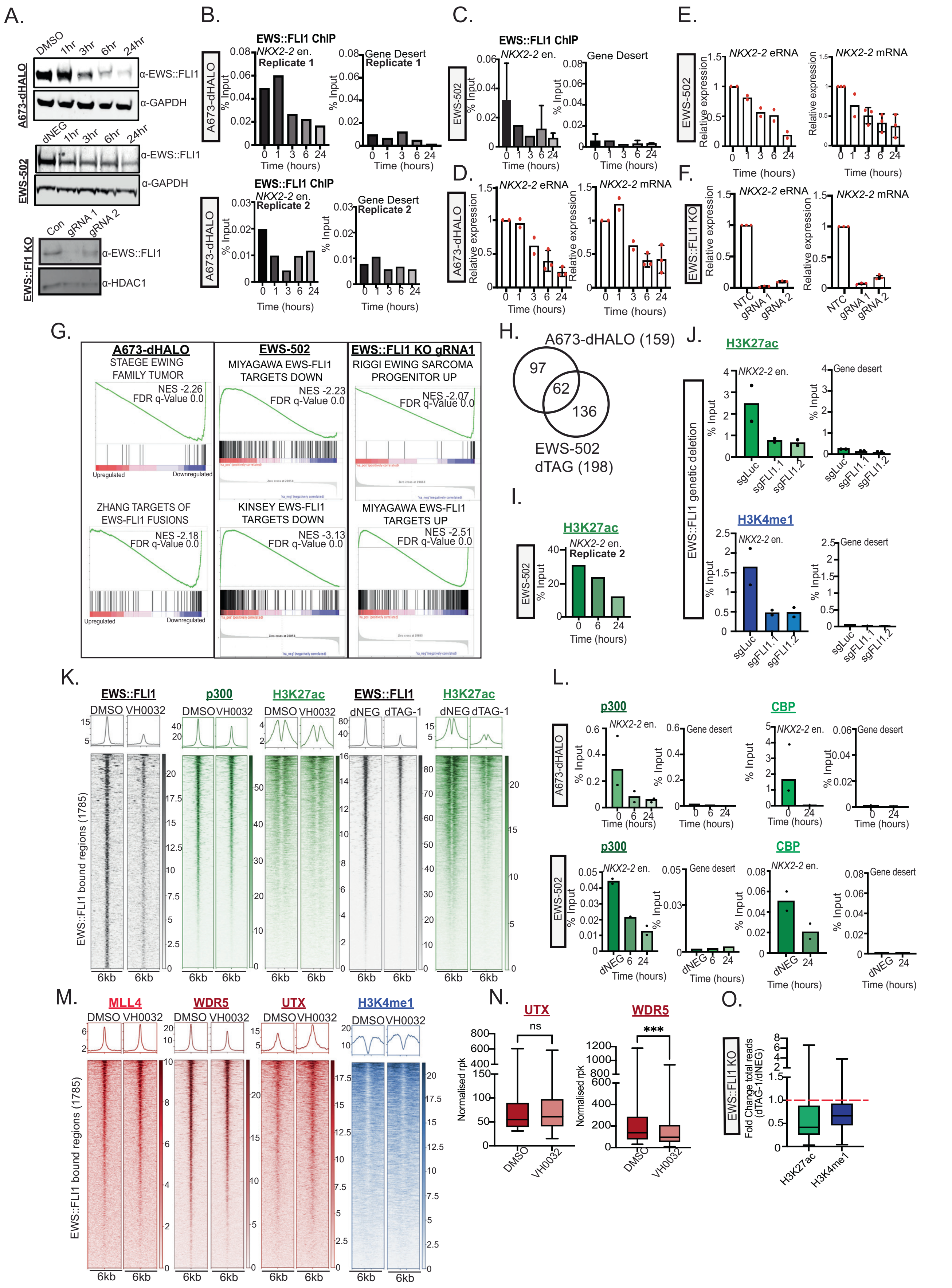
A.
DMSO 1hr 3hr 6hr 24hr
A673-dHALO
α-EWS::FLI1
α-GAPDH
dNEG 1hr 3hr 6hr 24hr
EWS-502
α-EWS::FLI1
α-GAPDH
Con gRNA 1 gRNA 2
EWS::FI1 KO
α-EWS::FLI1
α-HDAC1
B.
EWS::FLI1 ChIP
A673-dHALO
NKX2-2 en. Replicate 1
Gene Desert Replicate 1
NKX2-2 en. Replicate 2
Gene Desert Replicate 2
% Input
Time (hours)
C.
EWS::FLI1 ChIP
EWS-502
NKX2-2 en.
Gene Desert
D.
A673-dHALO
NKX2-2 eRNA
NKX2-2 mRNA
Relative expression
E.
EWS-502
NKX2-2 eRNA
NKX2-2 mRNA
F.
EWS::FLI1 KO
NKX2-2 eRNA
NKX2-2 mRNA
NTC gRNA 1 gRNA 2
G.
A673-dHALO
STAEGE EWING FAMILY TUMOR
NES -2.26 FDR q-Value 0.0
EWS-502
MIYAGAWA EWS-FLI1 TARGETS DOWN
NES -2.23 FDR q-Value 0.0
EWS::FLI1 KO gRNA1
RIGGI EWING SARCOMA PROGENITOR UP
NES -2.07 FDR q-Value 0.0
ZHANG TARGETS OF EWS-FLI1 FUSIONS
NES -2.18 FDR q-Value 0.0
KINSEY EWS-FLI1 TARGETS DOWN
NES -3.13 FDR q-Value 0.0
MIYAGAWA EWS-FLI1 TARGETS UP
NES -2.51 FDR q-Value 0.0
Upregulated
Downregulated
H.
A673-dHALO (159)
97
62
136
EWS-502 dTAG (198)
I.
H3K27ac
EWS-502
NKX2-2 en. Replicate 2
% Input
Time (hours)
J.
EWS::FLI1 genetic deletion
H3K27ac
NKX2-2 en.
Gene desert
H3K4me1
sgLuc sgFLI1.1 sgFLI1.2
K.
EWS::FLI1
p300
H3K27ac
DMSO VH0032
dNEG dTAG-1
EWS::FLI1 bound regions (1785)
6kb
L.
A673-dHALO
p300
CBP
NKX2-2 en.
Gene desert
EWS-502
Time (hours)
M.
MLL4
WDR5
UTX
H3K4me1
DMSO VH0032
EWS::FLI1 bound regions (1785)
6kb
N.
UTX
ns
WDR5
***
Normalised rpk
DMSO VH0032
O.
EWS::FLI1 KO
Fold Change total reads (dTAG-1/dNEG)
H3K27ac H3K4me1

Figure EV2. Rapid degradation of EWS::FLI1 is acutely linked to reduced p300 occupancy and H3K27ac.

(**A**) FLI1 western blot analysis of in A673-dHALO cells following 1 μM VH0032 treatment, EWS-502 dTAG cells following 1 μM dTAG-1 treatment and A673 cells following FLI1 genetic deletion for 7 days. (**B**) FLI1 ChIP-qPCR duplicates from two independent experiments in A673-dHALO cells following 1 μM VH0032 treatment. (**C**) FLI1 ChIP-qPCR in EWS-502 dTAG cells following 1 μM dTAG-1 treatment from two biological replicates. (**D–F**) RT-qPCR in (**D**) A673-dHALO cells following 1 μM VH0032 treatment. (**E**) EWS-502 dTAG system following 1 μM dTAG-1 treatment and (**F**) RT-qPCR in A673 cells following 7 days EWS::FLI1 knockout using two independent gRNAs. Error bars represent standard error of the mean from three biological replicates. (**G**) GSEA plots showing negatively enriched EWS::FLI1 gene expression signatures in A673-dHALO VH0032 treated cells, EWS-502 dTAG-1 treated cells and EWS::FLI1 KO in A673 cells. (**H**) Overlap of downregulated EWS::FLI1-sensitive gene targets between A673-dHALO and EWS-502 dTAG degrader cell lines. (**I**) H3K27ac ChIP-qPCR replicate 2 following 6- and 24 h of EWS::FLI1 degradation in EWS-502 dTAG system (for replicate 1 see Fig. 2). (**J**) H3K27ac and H3K4me1 ChIP-qPCR following 7 days EWS::FLI1 genetic deletion from two independent biological replicates. (**K**) Tornado plots showing p300, FLI1 and H3K27ac ChIP-seq signal at EWS::FLI1 enhancers in A673-dHALO and EWS-502 dTAG systems following 24 h degradation. A673-dHALO control plots as in Fig. 1A,B. (**L**) p300 and CBP ChIP-qPCR after 24 h degradation in both degrader settings from at least two biological replicates. (**M**) MLL4, WDR5, UTX and H3K4me1 ChIP-seq signal at EWS::FLI1 enhancers after 24 h 1 μM VH0032 treatment in A673-dHALO system. A673-dHALO control plots as in Fig. 1A,B. (**N**) UTX and WDR5 normalized rpk at EWS::FLI1-sensitive enhancers in A673-dHALO system. Mann–Whitney *U* test, ns $P = 0.382$, ***$P = 0.0005$. Boxes depict the range between the first and third quartile. Central line within box shows the median value and whiskers highlight the maximum to minimum data points. ChIP-seq from one biological replicate. (**O**) Fold change in H3K27ac and H3K4me1 ChIP-seq signal at EWS::FLI1 enhancers following EWS::FLI1 KO in A673 cells. Red line depicts steady-state levels. Boxes depict the range between the first and third quartile. Central line within box shows the median value and whiskers highlight the maximum to minimum data points. ChIP-seq from one biological replicate.

 © The Author(s)

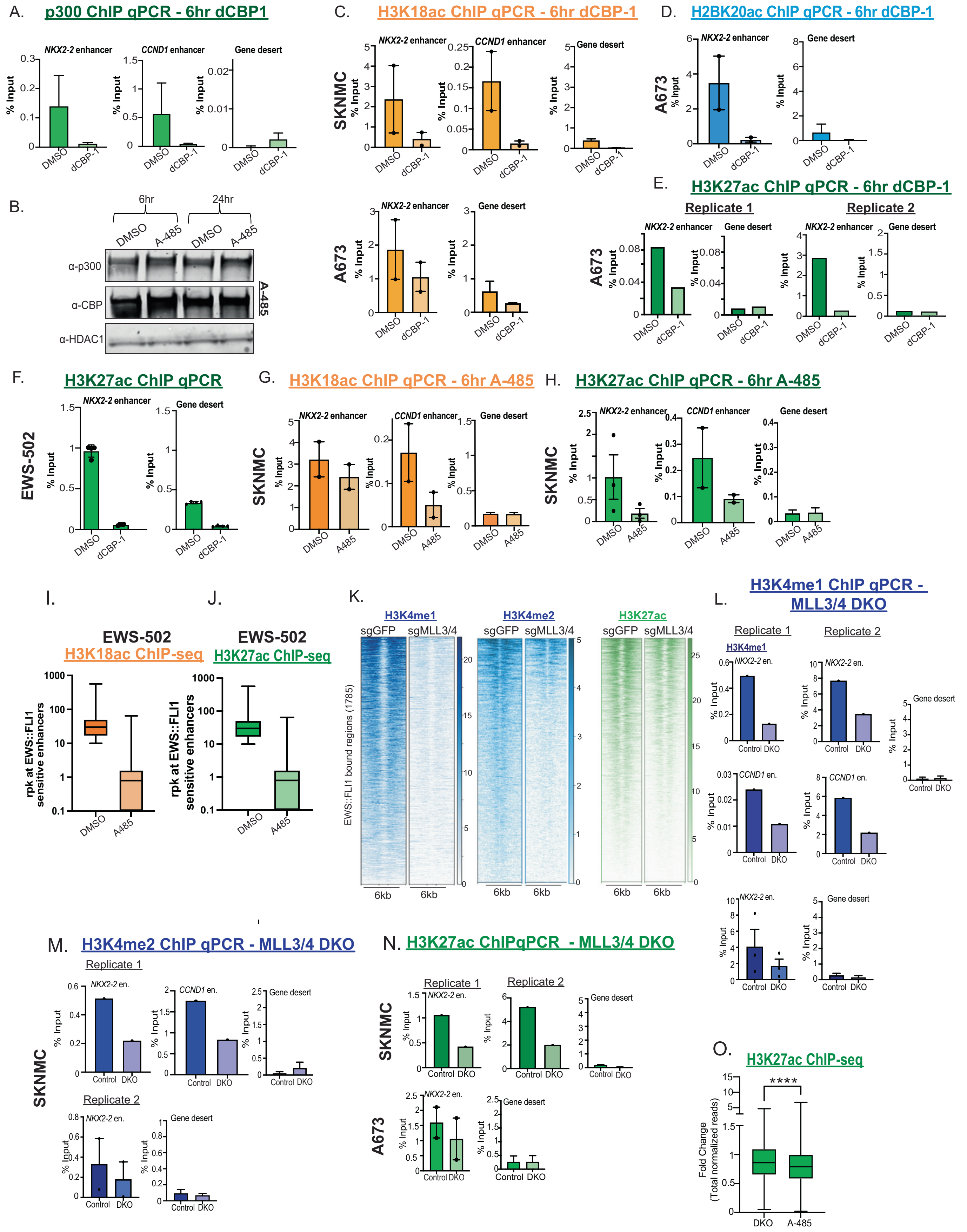
A. p300 ChIP qPCR - 6hr dCBP1
NKX2-2 enhancer
CCND1 enhancer
Gene desert
% Input
DMSO
dCBP-1
B.
6hr
24hr
DMSO
A-485
α-p300
α-CBP
α-HDAC1
A-485
C. H3K18ac ChIP qPCR - 6hr dCBP-1
SKNMC
A673
D. H2BK20ac ChIP qPCR - 6hr dCBP-1
E. H3K27ac ChIP qPCR - 6hr dCBP-1
Replicate 1
Replicate 2
F. H3K27ac ChIP qPCR
EWS-502
G. H3K18ac ChIP qPCR - 6hr A-485
H. H3K27ac ChIP qPCR - 6hr A-485
A485
I.
EWS-502
H3K18ac ChIP-seq
J.
EWS-502
H3K27ac ChIP-seq
rpk at EWS::FLI1 sensitive enhancers
K.
H3K4me1
H3K4me2
H3K27ac
sgGFP
sgMLL3/4
EWS::FLI1 bound regions (1785)
6kb
L. H3K4me1 ChIP qPCR - MLL3/4 DKO
Control
DKO
M. H3K4me2 ChIP qPCR - MLL3/4 DKO
N. H3K27ac ChIPqPCR - MLL3/4 DKO
O. H3K27ac ChIP-seq
****
Fold Change (Total normalized reads)
DKO
A-485

Figure EV3. Loss of p300/CBP and MLL3/4 protein function alter the EWS::FLI1 enhancer histone modification landscape.

(**A**) p300 ChIP-qPCR in DMSO control and dCBP-1 conditions in SKNMC cells from two biological replicates. (**B**) p300 and CBP western blot from nuclear extracted SKNMC cells treated with A-485 for 6 h and 24 h. (**C**) H3K18ac ChIP-qPCR in SKNMC and A673 following 6 h dCBP-1 treatment from two biological replicates. (**D**, **E**) H2BK20ac and H3K27ac ChIP-qPCR at NKX2-2 enhancer in SKNMC and A673 following 6 h dCBP-1 treatment. Graphs shown for two biological replicates. (**F**) H3K27ac ChIP-qPCR at NKX2-2 enhancer following dCBP-1 (6 h) treatment from at least three biological replicates. (**G**, **H**) H3K18ac and H3K27ac ChIP-qPCR at *NKX2-2* and *CCND1* enhancer in SKNMC cells following 6 h A-485 treatment from at least two biological replicates. Error bars where present depict standard error of the mean from three biological replicates. (**I**) H3K18ac normalized rpk at EWS::FLI1 enhancers in DMSO and A-485 (6 h) condition in EWS-502 cells. ChIP-seq from one biological replicate. (**J**) H3K27ac normalized rpk at EWS::FLI1 enhancers in DMSO and A-485 (6 h) condition in EWS-502 cells. ChIP-seq from one biological replicate. (**K**) Histone modifications at EWS::FLI1 enhancers in sgGFP control and sgMLL3/4 DKO conditions in A673 cells. (**L**) H3K4me1 ChIP-qPCR in sgGFP control and sgMLL3/4 DKO conditions in SKNMC and A673 cells from two biological replicates. (**M**) H3K4me2 ChIP-qPCR in sgGFP control and sgMLL3/4 DKO conditions in SKNMC cells from two biological replicates. (**N**) H3K27ac ChIP-qPCR in sgGFP control and sgMLL3/4 DKO conditions in SKNMC and A673 cells from two biological replicates. (**O**) H3K27ac ChIP-seq fold change at all EWS::FLI1 enhancers in MLL3/4 DKO and A-485 (6 h) treatment compared to DMSO control in SKNMC cells. Kruskal–Wallis test, ****$P < 0.0001$. Boxes depict the range between the first and third quartile. Central line within box shows the median value and whiskers highlight the maximum to minimum data points. ChIP-seq from one biological replicate.

© The Author(s)

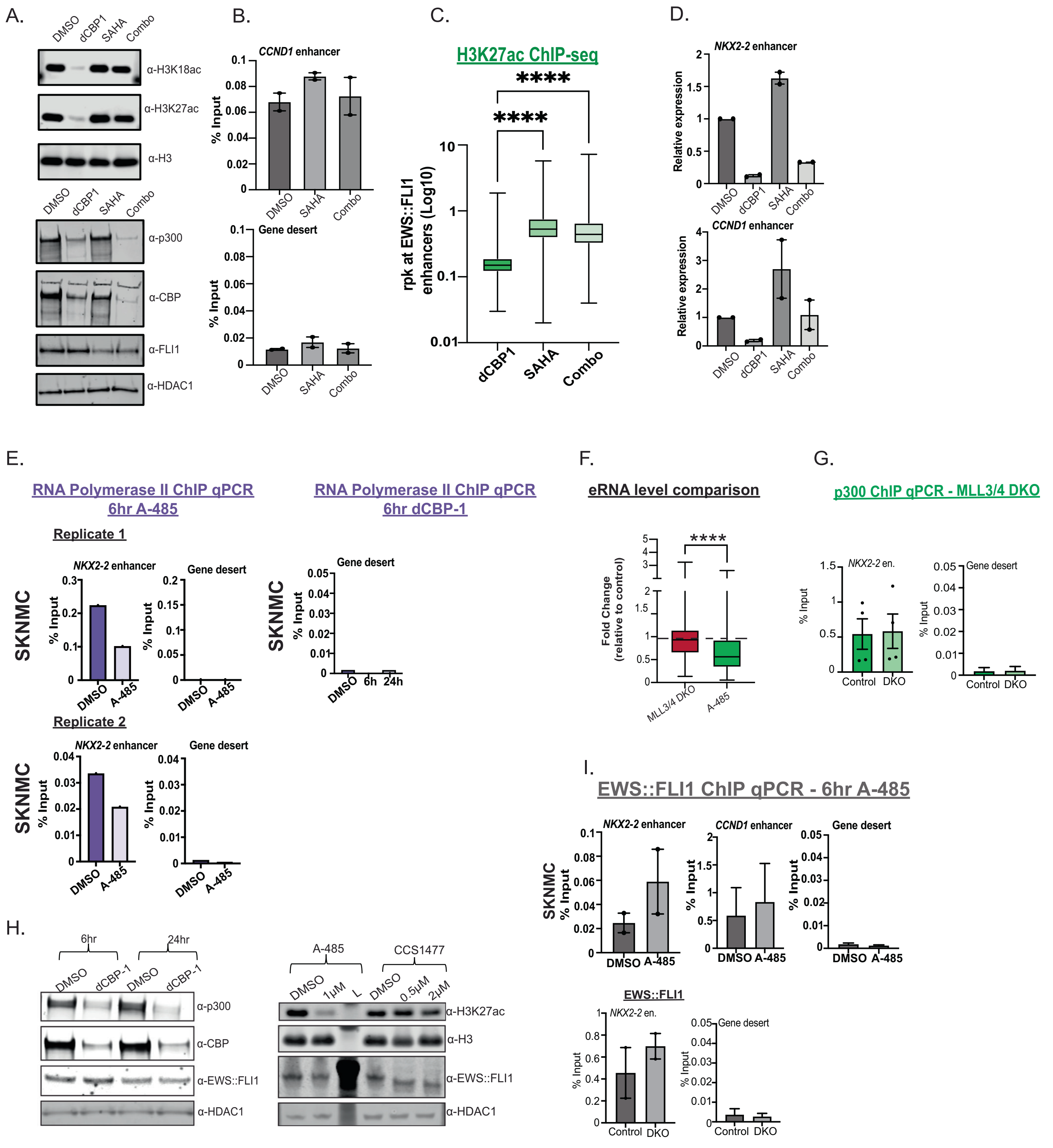
A.
DMSO
dCBP1
SAHA
Combo
α-H3K18ac
α-H3K27ac
α-H3
α-p300
α-CBP
α-FLI1
α-HDAC1
B.
CCND1 enhancer
% Input
Gene desert
C.
H3K27ac ChIP-seq
****
****
rpk at EWS::FLI1 enhancers (Log10)
D.
NKX2-2 enhancer
Relative expression
CCND1 enhancer
E.
RNA Polymerase II ChIP qPCR 6hr A-485
RNA Polymerase II ChIP qPCR 6hr dCBP-1
Replicate 1
Replicate 2
SKNMC
A-485
6h
24h
F.
eRNA level comparison
Fold Change (relative to control)
MLL3/4 DKO
G.
p300 ChIP qPCR - MLL3/4 DKO
NKX2-2 en.
Control
DKO
H.
6hr
24hr
dCBP-1
α-EWS::FLI1
CCS1477
I.
EWS::FLI1 ChIP qPCR - 6hr A-485
EWS::FLI1

Figure EV4. p300/CBP is required for enhancer RNA levels and RNA polymerase II occupancy at EWS::FLI1 enhancers independently of EWS::FLI1 binding.

(**A**) Western blot analysis in dCBP-1, SAHA and combined SAHA and dCBP-1 treatment. (**B**) FLI1 ChIP-qPCR in DMSO, SAHA and combined SAHA and dCBP-1 treatment. Graphs depict two biological replicates. (**C**) H3K27ac ChIP-seq normalized rpk at EWS::FLI1 enhancers in dCBP-1, SAHA and combined SAHA and dCBP-1 treatment. Boxes depict the range between the first and third quartile. Central line within box shows the median value and whiskers highlight the maximum to minimum data points. ChIP-seq from one biological replicate. (**D**) RT-qPCR at EWS::FLI1 enhancers in SKNMC cells following 24 h treatment of either 100 nM dCBP-1, 1 μM SAHA or dual treatment from two biological replicates. (**E**) RNA polymerase II ChIP-qPCR at *NKX2-2* enhancer in SKNMC cells following 6 h A-485 and 6 h dCBP-1 treatment from two biological replicates. (**F**) Fold change in eRNA RNA-seq reads at EWS::FLI1-sensitive enhancers following MLL3/4 DKO compared to A-485 treatment. Mann–Whitney *U* test, ****$P < 0.0001$. Boxes depict the range between the first and third quartile. Central line within box shows the median value and whiskers highlight the maximum to minimum data points. eRNA expression calculated from three biological replicates. (**G**) p300 ChIP-qPCR in sgGFP control and sgMLL3/4 DKO conditions in A673 cells. Error bars represent standard error of the mean from at least three biological replicates. (**H**) Western blot analysis of EWS::FLI1 from nuclear extracts of SKNMC cells treated with dCBP-1, A-485 or CCS1477. 'L' indicates protein ladder lane. (**I**) EWS::FLI1 ChIP-qPCR at *NKX2-2* and *CCND1* enhancers in SKNMC cells following 6 h A-485 treatment from two biological replicates.

 © The Author(s)

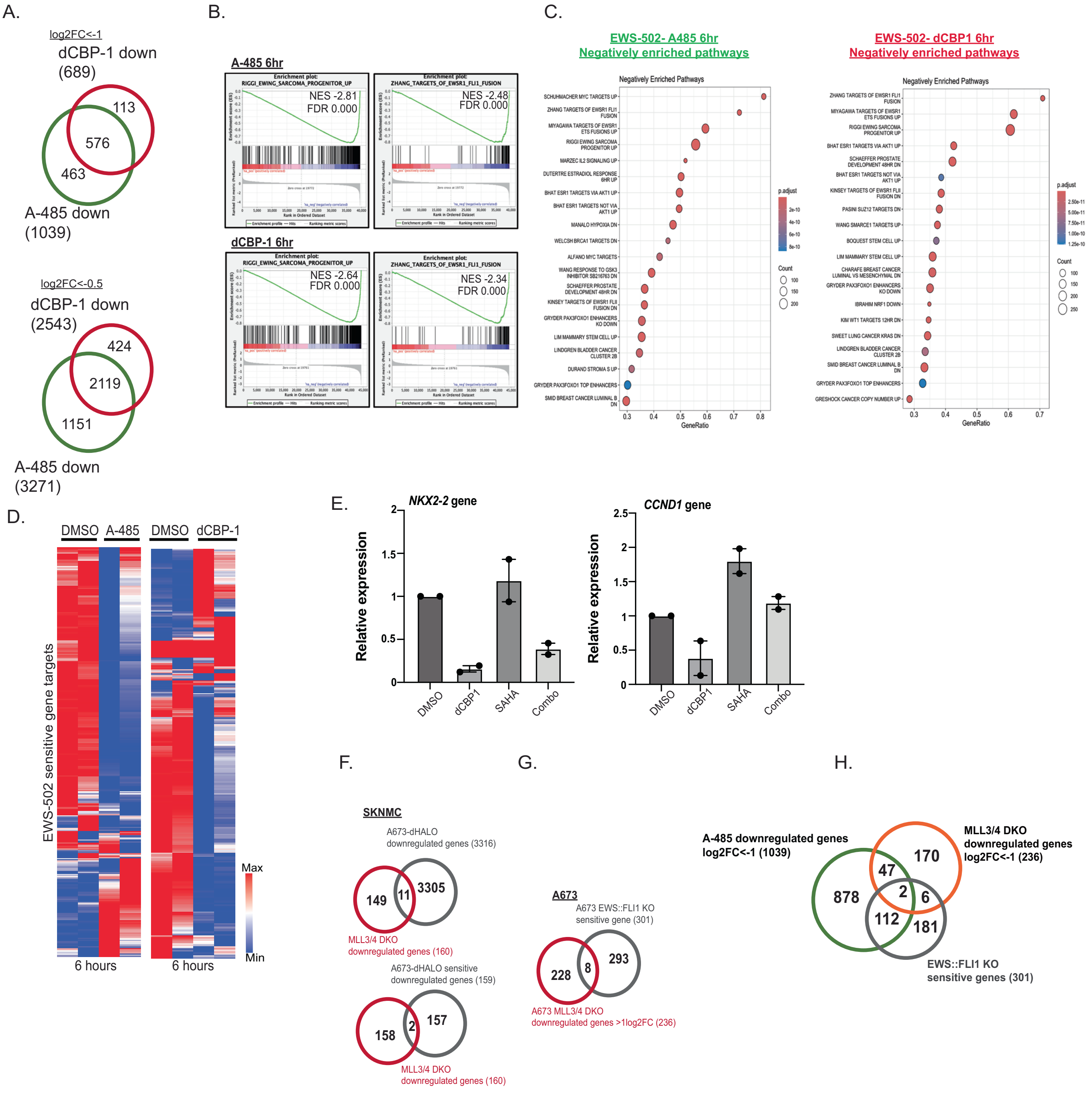

Figure EV5. p300/CBP function are central to the EWS::FLI1 gene regulatory network.

(**A**) Overlap between downregulated genes in A-485 and dCBP-1 conditions at two different log2FC cut off thresholds. (**B**) GSEA negatively enriched EWS::FLI1 signatures in both conditions. (**C**) Gene set enrichment scores showing top 20 negatively enriched gene sets following 6 h A-485 and 6 h dCBP-1 treatment, in order of enrichment in EWS-502 cells. Color represents *P*.adjust values from a Benjamini-Hochberg (BH) procedure test. Gene Ratio represents total differentially expressed genes in given gene set. (**D**) Z-scores of EWS::FLI1-sensitive enhancers and genes in DMSO, dCBP-1 and A-485 treatment conditions. (**E**) RT-qPCR at EWS::FLI1 genes in SKNMC cells following 24 h treatment of either 100 nM dCBP-1, 1 μM SAHA or dual treatment from two biological replicates. (**F**) Overlap between MLL3/4 DKO downregulated genes in SKNMC cells with genes downregulated in A673-dHALO setting and EWS::FLI1-sensitive genes. (**G**) Overlap between MLL3/4 DKO downregulated genes in SKNMC cells with genes downregulated in EWS::FLI1 KO setting in A673 cells. (**H**) Overlap between A-485 downregulated genes (either log2FC < -1 or log2FC < −0.5), MLL3/4 DKO downregulated genes (either log2FC < −1 or log2FC < -0.5) and EWS::FLI1 KO sensitive genes.

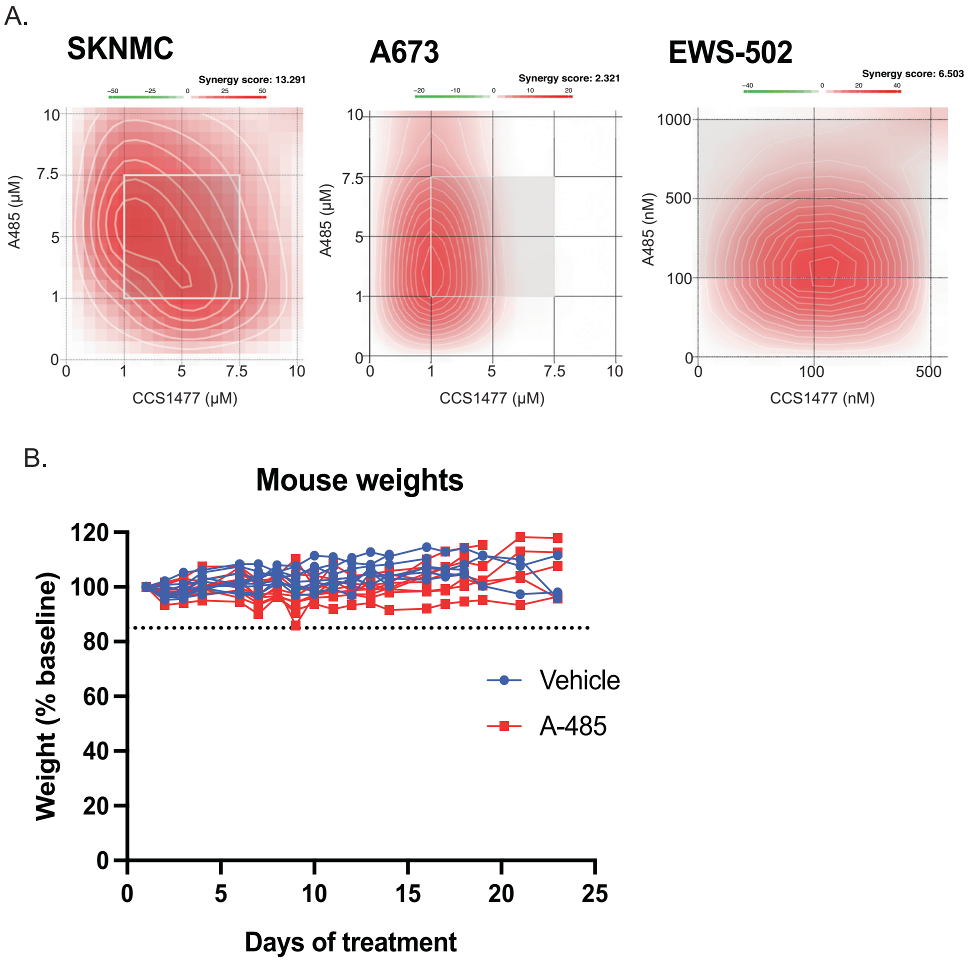

Figure EV6. p300/CBP inhibition decelerates Ewing sarcoma tumor growth in vivo.

(**A**) Synergy plots for combination treatment of A-485 and CCS1477 in A673, SKNMC and EWS-502 cells. (**B**) Percentage weight of mice compared to day 1 of treatment in vehicle-only (blue) and 100 mg/kg A-485 (red) cohorts. Each point and line represent a single mouse.

 © The Author(s)
